# Polymorphic Variants of Genes Encoding Angiogenesis-Related Factors in Infertile Women with Recurrent Implantation Failure

**DOI:** 10.3390/ijms24054267

**Published:** 2023-02-21

**Authors:** Aleksandra E. Mrozikiewicz, Grażyna Kurzawińska, Marcin Ożarowski, Michał Walczak, Katarzyna Ożegowska, Piotr Jędrzejczak

**Affiliations:** 1 Department of Obstetrics and Women’s Diseases, Poznan University of Medical Sciences, Polna 33, 60-535 Poznan, Poland; 2 Chair and Department of Cell Biology, Poznan University of Medical Sciences, Rokietnicka 5D, 60-806 Poznan, Poland; 3 Division of Perinatology and Womens Diseases, Poznan University of Medical Sciences, Polna 33, 60-535 Poznan, Poland; 4 Department of Biotechnology, Institute of Natural Fibres and Medicinal Plants—National Research Institute, Wojska Polskiego 71B, 60-630 Poznan, Poland; 5 Institute of Human Genetics, Polish Academy of Sciences, Strzeszyńska 32, 60-479 Poznan, Poland; 6 Department of Infertility and Reproductive Endocrinology, Poznan University of Medical Sciences, Polna 33, 60-535 Poznan, Poland

**Keywords:** RIF, ART, IVF, SNP, *VEGFA*, *FGF2*, *FLT1*, KDR

## Abstract

Recurrent implantation failure (RIF) is a global health issue affecting a significant number of infertile women who undergo in vitro fertilization (IVF) cycles. Extensive vasculogenesis and angiogenesis occur in both maternal and fetal placental tissues, and vascular endothelial growth factor (VEGF) and fibroblast growth factor (FGF) family molecules and their receptors are potent angiogenic mediators in the placenta. Five single nucleotide polymorphisms (SNPs) in the genes encoding angiogenesis-related factors were selected and genotyped in 247 women who had undergone the ART procedure and 120 healthy controls. Genotyping was conducted by polymerase chain reaction-restriction fragment length polymorphism (PCR-RFLP). A variant of the kinase insertion domain receptor (*KDR*) gene (rs2071559) was associated with an increased risk of infertility after adjusting for age and BMI (OR = 0.64; 95% CI: 0.45–0.91, *p* = 0.013 in a log-additive model). Vascular endothelial growth factor A (*VEGFA*) rs699947 was associated with an increased risk of recurrent implantation failures under a dominant (OR = 2.34; 95% CI: 1.11–4.94, p_adj._ = 0.022) and a log-additive model (OR = 0.65; 95% CI 0.43–0.99, p_adj._ = 0.038). Variants of the *KDR* gene (rs1870377, rs2071559) in the whole group were in linkage equilibrium (D’ = 0.25, r^2^ = 0.025). Gene–gene interaction analysis showed the strongest interactions between the *KDR* gene SNPs rs2071559–rs1870377 (*p* = 0.004) and *KDR* rs1870377–*VEGFA* rs699947 (*p* = 0.030). Our study revealed that the *KDR* gene rs2071559 variant may be associated with infertility and rs699947 *VEGFA* with an increased risk of recurrent implantation failures in infertile ART treated Polish women.

## 1. Introduction

Recurrent implantation failure (RIF) is the condition in which the embryo fails to implant after at least three transfers in three consecutive in vitro fertilization (IVF) cycles. Currently, RIF is considered one of the main challenges of reproductive medicine and concerns about 15% of women treated for infertility [1]. Moreover, it was estimated that 5% of women suffer from recurrent pregnancy loss, 75% of cases of which were observed to be due to RIF [1]. The described risk factors of RIF include advanced maternal age, BMI, tobacco, alcohol intake, and endometriosis. The reasons for RIF could also be divided into embryo factors (genetic abnormalities) and uterine factors (anatomical abnormalities; immunological factors; biomolecular factors; glycodelin-A; infection). However, the influence of the male factor (sperm quality) and female factors (low quality of gametes; thrombophilia; inherited and acquired; other genetic polymorphisms such as miRNA, HLA–G, p53, VEGF; vitamin D deficiency; alterations in vaginal microbiota) on the occurrence of RIF has also been observed [2,3,4]. Unfortunately, the complex details of the processes that occur in women with RIF remain unclear to date. Moreover, recurrent pregnancy loss (RPL) is multifactorial and many cases remain unexplained. Recurrent implantation failures and recurrent miscarriages has partially overlapping causes, and the association of genetic variants with RPL is more frequently studied [5,6,7]. In both RIF and RPL, the problem is pregnancy loss, but women with RIF also have difficulty getting pregnant. Therefore, despite the similarities, the causes of RIF and RPL may differ.

Implantation is essential for embryo survival and successful reproduction. This process requires the competent blastocyst, receptive endometrium, and the synchronized dialogue between maternal and embryonic tissues. The delicate balance between these factors is very important for the embryo adhesion and attachment to the endometrium and the formation of fetal–mother contact [8,9,10]. In the next phase, the embryo invades the endometrium and blood cells arise from the mesoderm. A normal pregnancy requires the development of the complex vascular network of both the mother and the fetus to meet the increasing oxygen and metabolic demands of the developing embryo. The placenta is a unique vascular organ that receives blood supplies from both the maternal and the fetal systems and thus has two separate blood circulatory systems [11].

Blood vessels form in two ways: vasculogenesis, whereby vessels arise from blood islands, and angiogenesis (branching and nonbranching), which entails sprouting from existing vessels [12]. Extensive angiogenesis occurs in both the maternal and fetal placental tissues [13]. The embryonic vasculature is formed by the segregation, migration and assembly of mesodermal angioblasts, a process called vasculogenesis. In the complex process of angiogenesis, the activity of many growth factors and their receptors on various pathways plays a key role. The most potent angiogenic factors to promote vasculogenesis and angiogenesis in the placenta include vascular endothelial growth factors (VEGFs) and their receptors (VEGFRs), FGF family molecules, the angiopoietin system, and many others [11]. Angiogenesis is a multi-stage process, during which significant changes occur in the environment surrounding the cells. Growth factors increase vascular permeability, stimulate specific proteases (collagenases and plasminogen activators) to proteolytic degradation of the extracellular matrix (ECM) and cause proliferation of endothelial cells. The final stage is followed by chemotactic migration of endothelial cells and invasion of the ECM, formation of the lumen and functional maturation of the endothelium [14].

In humans, the VEGF family is composed of multiple isoforms encoded by five genes (*VEGFA*, *VEGFB*, *VEGFC*, *VEGFD*, and placental growth factor—*PIGF*). These ligands bind to VEGFRs belong to the type IV receptor tyrosine kinase (RTK) family and include VEGFR1 (*FLT1* gene), VEGFR2 (*KDR* gene) and VEGFR3 (*FLT4* gene). VEGFR1 and VEGFR2 regulate angiogenesis and vascular permeability, and VEGFR3 mainly regulates lymphangiogenesis [15]. VEGF also interacts with heparan sulfate proteoglycans (HSP), and neuropilin 1 and 2 co-receptors (NRP1 and NRP2). Moreover, growth factors are dimers and can form both homo and heterodimers [16].

*VEGFA* (usually called *VEGF*), first described by Senger et al. [17], is one of the most studied growth factors. It is a highly specific vascular endothelial cell mitogen and also the strongest pro-angiogenic factor in the VEGF family. *VEGFA* binds with high affinity to two VEGF receptor tyrosine kinases (VEGFR1, VEGFR2) and with lower affinity to co-receptors NRP1 and NRP2 [16,18,19]. There is a correlation between altered VEGF expression and reproductive failure, including recurrent implantation failure and recurrent miscarriage (RM) [20].

The *VEGFA* gene is highly polymorphic, especially in the promoter, 5′-untranslated and 3′-untranslated regions. Some of these variants—rs699947 (−2578C > A), rs1570360 (−1154G > A), rs2010963 (−634C > G) and rs3025039 (+936C > T)—have been associated with variable VEGF protein expression and serum VEGFA levels [21]. Several publications have reported an association of *VEGFA* gene variants rs833061 (−460T > C), rs25648 (−7C > T) and mainly rs1570360 (−1154G > A) with recurrent implantation failures [22,23,24,25].

As well as the expression of angiogenic factors during embryonic implantation, also the expression of their receptors has been demonstrated. In the placenta, the activity of VEGFR1 and VFGFR2 receptors was observed [26,27]. Tyrosine kinase 1 (FLT1) is the VEGFA and placental growth factor receptor and is expressed in the trophoblasts of the placenta throughout gestation. A soluble form of VEGFR1 called sFlt-1 is markedly increased during the last two months of preeclamptic pregnancy compared with normotensive pregnant controls [28]. The rs722503 polymorphism is located in intron 10 of the *FLT1* gene and can alter the regulatory motif for binding of nuclear factor-κB (NF-κB). NF-κB is a transcription factor that can participate in both activation and repression of transcription and is associated with angiogenesis and cell proliferation [29,30,31]. In addition, multiple-SNP analysis by Wujcicka et al. [32] showed that the TT variants for *CSF2* (rs25881) and *FLT1* (rs722503) polymorphisms were associated with an approximately two-fold increase in the prelabor rupture of membranes (PROM) risk when corrected for APTT and PLT parameters and pregnancy.

The kinase insertion domain receptor (KDR), also known as vascular endothelial growth factor receptor 2 (VEGFR2), plays an important role in embryonic development. VEGF-activated receptor stimulates endothelial cell proliferation and is crucial for the development of the embryonic vascular system and hematopoietic system [33,34]. Studies show that the minor allele G of the rs2071559 polymorphism, located in the promoter region, may lead to a decrease in VEGFR2 transcriptional activity, while the minor allele T of the rs1870377 (Gln472His) polymorphism has been associated with reduced VEGFR2 binding affinity [35,36].

Basic fibroblast growth factor 2 (FGF2) is the prototype member of a family of structurally related fibroblast growth factors (FGFs). Growing evidence suggests that fibroblast growth factor/FGF receptor (FGF/FGFR) signaling has crucial roles in a multitude of processes during embryonic development and adult homeostasis by regulating cellular lineage commitment, differentiation, proliferation, and apoptosis of various types of cells. Fibroblast growth factor 2 (FGF2) has a particular role in the formation of endothelial precursors, angioblasts, and their assembly into the initial pattern of the vasculature early during embryonic development [37,38,39]. The rs308395 polymorphism within the *FGF2* gene promoter may influence transcription factor binding, and thus FGF2 expression [40].

Considering the above-mentioned interesting insights, we tested the hypothesis that single nucleotide polymorphisms (SNPs) in genes encoding the angiogenesis pathway predispose to infertility and recurrent implantation failure. We evaluated the association of five polymorphic variants in *VEFGA* (rs699947), *FLT1* (rs722503), *KDR* (rs2071559, rs1870377) and *FGF2* (rs308395) genes with infertility and recurrent implantation failure among Polish women.

## 2. Results

### 2.1. Baseline Characteristics of Study Subjects and Control Groups

There was no significant difference in maternal age between cases and controls (33.11 ± 3.51 vs. 32.50 ± 3.60 years, *p* = 0.123). Body mass index was significantly higher in the cases than in the control group (23.36 ± 4.17 vs. 20.71 ± 1.79, *p* < 0.001). Over a quarter (25.9%) of the women in the study group had a BMI above 25. In the cases, the median AMH before ART treatment level was 21.00 pmol/L (IQR 11.17–30.79).

Of the total 247 infertile women who underwent an ART treatment cycle, 70.9% had a maximum of two prior failed embryo transfers and 29.1% had at least three prior failed embryo transfers (RIF patients). In 89 cases, the indication for the ART procedure was the male factor, in 119 cases idiopathic infertility and in 39 cases the female factor (oviduct + ovulatory). In the study group, 95 (38.5%) women did not become pregnant. One hundred and twenty women (48.6%) achieved one, 11.3% two, and four women (1.6%) achieved three pregnancies. From the whole number of 188 pregnancies obtained after in vitro fertilization, in 55 cases (29.3%) fresh embryo transfer was performed and in 133 cases (70.7%) frozen embryo transfer was performed. Detailed patient characteristics are summarized in Table 1.

### 2.2. Association Studies

As a first step the frequencies of genotypes and alleles of selected *VEGFA*, *FLT1*, *KDR* and *FGF2* polymorphisms were analyzed. The genotype distribution of these SNPs in controls were in accordance with the Hardy–Weinberg equilibrium (*p* > 0.05). Differences in SNP allele frequency distribution between the cases and the healthy controls were analyzed using the chi^2^ test and odds ratios (ORs). A statistically significant difference was observed only for *KDR* rs2071559. Compared with the A allele, the G allele of rs2071559 was more frequent in infertile women (0.55% vs. 0.47% in controls, OR = 1.378, 95% CI 1.011–1.877, *p* = 0.042 (Table 2).

Multiple logistic regression analysis with adjustment for age and BMI was performed in codominant, dominant, recessive, over-dominant and log-additive models. The genotype distribution of these SNPs is shown in Table 3. Based on the data, *KDR* rs2071559 was associated with an increased risk of infertility in crude analysis under a log-additive model (major allele homozygotes vs. heterozygotes vs. minor allele homozygotes); *p* = 0.034. After adjusting for age, BMI was significantly associated under a codominant model (*p* = 0.04190), a recessive model (AA + AG vs. GG: OR = 1.89; 95% CI 1.07–3.34, *p* = 0.025) and a log-additive model (OR = 0.64; 95% CI 0.45–0.91, *p* = 0.013). The results indicated that rs2071559 might have a significant association with infertility in our population. For other analyzed polymorphisms, no statistically significant difference was observed (all *p* > 0.05) (Table 3).

### 2.3. Stratification Analysis

In order to investigate the possible impact of the analyzed SNPs on the occurrence of recurrent implantation failures, we divided 247 infertile cases into two subgroups: women with RIF (*n* = 72) and those with less than 3 previous failed embryo transfers (*n* = 175). Clinical characteristics are shown in Table 4. Comparing the groups separated in this way, we observed that the patients with RIF were statistically significantly older (mean ± SD: 34.1 ± 3.7 vs. 32.7 ± 3.4 years, *p* = 0.005). However, we did not observe any differences in BMI means and serum AMH level medians between groups. In both groups, the indications for the ART procedure and the type of embryos used were similar (*p* = 0.763 and *p* = 0.6985, respectively). As many as 58.3% of women with recurrent implantation failures never became pregnant. In the RIF group, 41 pregnancies were achieved, whereas in the group without RIF there were 147 (Table 4).

A comparison of pregnancy outcomes was also made between fresh embryos (*n* = 55) and frozen embryos (*n* = 133) after IVF treatment. Pregnancy ended with childbirth in 85.5% of mothers from the fresh and in 88.7% from the frozen embryo transfer. Women who underwent frozen blastocyst transfer more often gave birth by caesarean section (65.3% vs. 44.7%, *p* = 0.015). The average birth weight of infants was slightly lower in the fresh embryo transfer group and was 3315.1 ± 512.6 g compared to 3458.1 ± 412.8 g in the frozen group (*p* = 0.0940). There were no statistically significant differences between the groups in gestational age, placenta weight and Apgar score (Table 5).

Next, we evaluated the possible associations between studied polymorphic variants and recurrent implantation failures. Our data indicated no significant difference in the genotype frequencies of studied *FLT1*, *KDR* and *FGF2* gene polymorphisms between RIF and NO-RIF women.

However, comparing subgroups, we observed a statistically significant difference between them for the *VEGFA* rs699947 variant. In the codominant model, the genotype frequency was: CC–27.4% and 13.9%, CA–50.9% and 58.3%, AA–21.7% and 27.8% in women without and with RIF, respectively (*p* = 0.070, p_adj._ = 0.052). This SNP was associated with an increased risk of recurrent implantation failures under a dominant (OR = 2.34; 95% CI 1.11–4.94, *p* = 0.023, p_adj._ = 0.022) and a log-additive model (OR = 0.65; 95% CI 0.43–0.99, *p* = 0.040, p_adj._ = 0.038) (Table 6).

### 2.4. Haplotype and Gene–Gene Interaction Analysis

**Figure 1:**
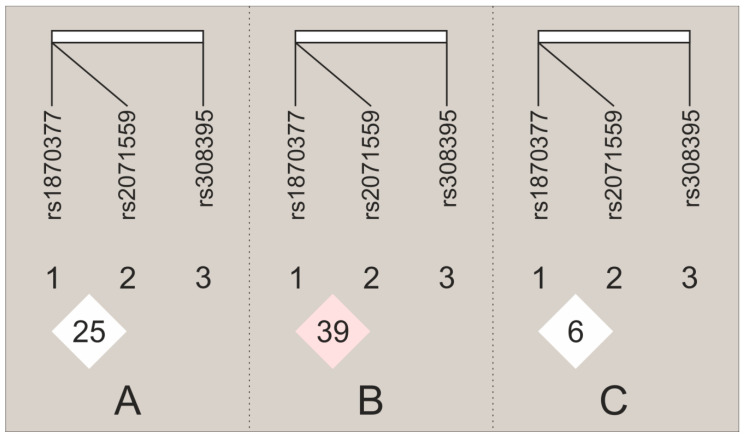
Linkage disequilibrium (LD) maps of the *KDR* and *FGF2* gene polymorphisms for all study women (**A**), cases (**B**) and controls (**C**). The map illustrates the pairwise LD between SNPs based on D′ values. The numbers in the triangles represent the D’ *100 between the adjacent SNPs.

To generate a linkage disequilibrium (LD) map, polymorphisms of the *KDR* gene (rs1870377, rs2071559) and *FGF2* rs308395 located on the same chromosome 4 were selected. An LD plot was constructed using combined genotype data from both groups of cases and controls (plot 1A), only cases (plot 1B) and only for controls (plot 1C) using the program HaploView, version 4.1. The LD analysis showed that rs1870377 and rs2071559 (distance between 19392 bp) in the whole group (cases and controls) were in linkage equilibrium (D’ = 0.25, r^2^ = 0.025, LOD = 1.74); thus, haplotype analysis was not conducted. We only observed weak LD between examined *KDR* gene polymorphisms in infertile cases (D’ = 0.395, r^2^ = 0.053, LOD = 2.31). The results are shown in Figure 1.

**Figure 2:**
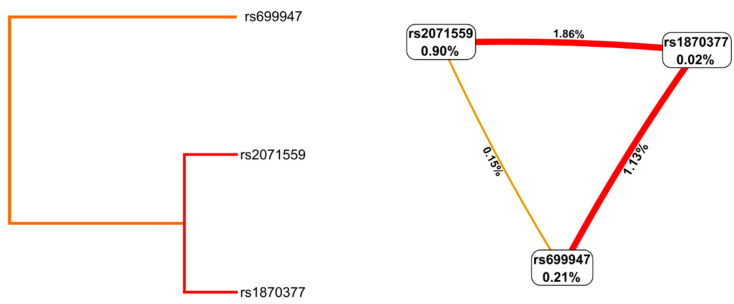
SNP–SNP interaction dendrogram and Fruchterman–Reingold plots obtained by multifactor dimensionality reduction for genes encoding angiogenesis related factors in infertility.

To search for gene–gene interactions, we used multifactor dimensionality reduction (MDR 3.0.2). Analysis of the dataset of infertile cases and controls revealed synergistic interactions between *KDR* rs2071559 and *KDR* rs1870377 (IG = 1.86%) and *KDR* rs1870377 and *VEGFA* rs699947 (IG = 1.13%) (Figure 2).

These gene relationships were confirmed in the SNPassoc package. The analysis showed the strongest interaction between the *KDR* gene rs2071559–rs1870377 (*p* = 0.004) and rs1870377–rs699947 (*p* = 0.030). The interaction between the polymorphism of the *KDR* rs2071559 gene and *VEGFA* rs699947 was not statistically significant (*p* = 0.372).

Statistical power for infertility susceptibility analysis was calculated by a Genetic Association Study (GAS) Power Calculator [41] using the following parameters. Numbers of cases and controls and allele frequencies are presented in Table 4 and Table 5. Infertility prevalence is 10–15% on average in the European populations [42]. Under an additive model, the power of our study to detect an association at a significance level of 0.05 was 10% (average for all tested SNPs) for a genotype relative risk (GRR) equal to 1.1 and 0.69% for a GRR 1.5.

## 3. Discussion

The proper development and function of the placenta are crucial not only for the survival and development of the fetus in utero. The placenta, being the first fetal organ to develop and to function normally, must be highly vascularized [13,43]. An appropriate course of angiogenesis is necessary for a successful pregnancy, and the correct uteroplacental circulation is crucial in the process of implantation and embryo development. Disruption of these processes can lead to various undesirable consequences in pregnancy, such as recurrent pregnancy loss, including recurrent miscarriage and recurrent implantation failure. Some of the most important genes involved in angiogenesis are from the vascular endothelial growth factor family. The best characterized family member is *VEGFA*, an important factor that regulates angiogenesis, with several isoforms, and that participates in multiple physiological pathways. Several polymorphisms have been reported in the promoter region of the *VEGFA* gene, including −2578C > A (rs699947) and −1154G > A (rs1570360), which are associated with altered VEGF secretion (Peach et al., 2018; Almawi et al., 2013). Several studies have been conducted in different populations to investigate the association between *VEGFA* gene polymorphisms and RIF, with conflicting results [22,23,24,25,44]. Most research between recurrent implantation failure and *VEGFA* gene polymorphisms has paid attention to the −1154G > A (rs1570360) variant. Although studies have been conducted in different populations, there is a noticeable relationship between RIF and the frequency of the minor −1154A allele. Turienzo et al. [22] reported that the rs1570360 polymorphism in the dominant model (GG vs. GA/AA) is associated with an increased risk of implantation failure (OR = 1.842, CI 95% 1.002–3.422). Goodman et al. [25] found that homozygosity of the *VEGFA* −1154AA gene was significantly higher among women experiencing recurrent implantation failure compared with fertile control women (19% vs. 5%, *p* = 0.02) and may serve as a susceptibility factor affecting the chances of recurrent implantation failure [25]. In addition, Vagnini et al. [23] found an association between this variant and RIF in Brazilian women (OR = 2.12 95% CI: 1.16–3.87, *p* = 0.01 in the dominant model). In a meta-analysis of three case–control studies comprising 305 RIF cases and 378 controls, Zeng et al. [45] confirmed the association of (−1154G > A) polymorphism and RIF under the allele (OR 1.39, 95% CI 1.08–1.78, *p* = 0.01) and dominant genetic model (OR 1.56, 95% CI 1.10–2.20, *p* = 0.01). Other polymorphic variants of the *VEGF* gene may also be associated with the occurrence of recurrent implantation failure. In 119 Korean women with RIF and 236 controls, the *VEGF* rs833061 (−460T > C), rs25648 (−7C > T) and rs3025020 (−583C > T) genetic polymorphisms were analyzed. The rs833061 C and rs25648 T *VEGF* alleles were associated with a higher risk of RIF (OR = 1.813, *p* = 0.009 and OR = 2.213, *p* = 0.005, respectively) [24]. Another study found that the *VEGF* rs2010963 (+405G > C in the 5′-untranslated region)  CC genotype may predispose to recurrent implantation failure after intracytoplasmic sperm injection—embryo transfer (ICSI-ET) [46]. In this study, we observed a statistically significant difference for *VEGFA* −2578C > A polymorphism between women without and with RIF. This variant was associated with an increased risk of recurrent implantation failures under a dominant (OR = 2.34; 95% CI: 1.11–4.94, *p* = 0.023, p_adj._ = 0.022) and a log-additive model (OR = 0.65; 95% CI: 0.43–0.99, *p* = 0.040, p_adj._ = 0.038). Although the polymorphism rs699947 selected in our work is very often studied in connection with various diseases, we found only one study that investigated the occurrence of RIF in Korean females. In the 116 women with RIF and 218 controls, the VEGF −2578C > A, −1154G > A, −634C > G and 936C > T genetic variants were determined. The VEGF -2578AA genotype was associated with an increased prevalence (≥4) of RIF (AOR = 2.77; 95% CI: 1.10–7.02; *p* = 0.031). The results of this research indicated that the VEGFA -2578AA genotype, −634G allele and −2578A/−1154A/−634G/936C haplotype could be a genetic marker of RIF. Interestingly, in this study, no statistically significant difference was observed between the RIF and the control women for the −1154G > A polymorphism [44].

The influence of *FLT1* gene polymorphisms is often studied in preeclampsia [29,30,47]. Soluble FLT1 (sFLT1), which is encoded by an alternatively spliced transcript of *FLT1*, is an antagonist of VEGF and PIGF. Levels of sFLT1 in maternal blood have been found to be elevated in PE patients. In white women, *FLT1* rs722503, *FLT4* rs307826, and *VEGFC* rs7664413 were significantly associated with preeclampsia [47]. Several studies have found circulating levels of sFLT1 to be raised in women with threatened abortion and RM [48,49]. However, little is known about the role of *FLT1* and its polymorphic variants in RIF. In a study by Bansal et al. [50], serum levels of VEGFA and its receptor FLT1 were compared with levels of NK cells, activated NK cells, and NK cytotoxicity in 62 women with re-implantation failure (RIF) and 72 healthy controls. VEGFA levels were found to be significantly elevated in women with RIF compared to healthy controls, but there was no difference in FLT1 levels between the groups. In our study, the *FLT1* gene rs722503 polymorphism was not associated with infertility or RIF in the population of Polish women. Genetic variants of the second VEGF receptor, encoded by the *KDR* gene, are a frequent subject of association studies with recurrent miscarriages. Rah et al. [51] reported that the kinase insert domain-containing receptor gene (−604T > C) rs2071559 polymorphism was associated with recurrent pregnancy loss in Korean women. In the present study, this variant was associated with an increased risk of infertility (after adjusting for age and BMI, rs2071559 was significantly associated under a codominant [*p* = 0.042], a recessive [*p* = 0.0245] and a log-additive model [*p* = 0.013]). For the second analyzed *KDR* polymorphism (rs1870377), no statistically significant difference was observed. However, in gene–gene interaction analysis, this variant was in strong interaction with VEGFA rs699947 (*p* = 0.030).

Fibroblast growth factor 2 (*FGF2*) belongs to the FGF superfamily, comprising at least 22 members in humans. It is a pleiotropic signaling molecule involved in many biological processes including angiogenesis, embryonic development and wound healing. FGF2 is widely used in stem cell research as an agent of self-renewal (proliferation) and differentiation in vitro [52]. Several polymorphisms in the *FGF2* gene have been identified, of which rs2922979 (intron), rs308395 (promoter) rs1476217 (3′-UTR), rs308397 (promoter), and rs3747676 (3′-UTR) are the most investigated. The rs308395 variant selected for this study was previously studied in connection with the development of high myopia, diabetic retinopathy, multiple myeloma, risk of cleft lip or in the process of restenosis in patients with stable coronary artery disease treated with a metal stent [53,54,55,56,57]. We did not observe an association of this SNP with infertility or recurrent implantation failures in the studied population of Polish women.

Our results show that the maternal body mass index was significantly higher in the infertile women than in the control group (23.36 ± 4.17 vs. 20.71 ± 1.79, *p* < 0.001). More than a quarter (25.9%) of women undergoing ART therapy were obese, which may indicate the importance of BMI in infertility. However, we did not observe differences in BMI means between the RIF groups and women with less than three previous failed embryo transfers. Recently, two interesting studies on this topic have been published. In the first, Nogales et al. (2021), in a multicenter study with 2832 patients undergoing pre-implantation genetic testing for aneuploidies (PGT-A), investigated which factors, excluding embryo aneuploidies, are associated with miscarriage in patients who have undergone a single euploid blastocyst transfer. One of the main findings was a significant relationship between body mass index (BMI) and miscarriage rates (13.4% in underweight women, 12.1% in normal weight, 14.5% in overweight, and 19.2% in obese women, odds ratio (OD) 1.04; 95% CI, 1.01–1.07, *p* = 0.006). However, in the second, Canadian study, gestational carriers (healthy women with proven fertility and a good obstetric history, who chose to carry a baby not genetically related to them for intended parents) were matched by BMI to infertile patients treated during the same years provided they had undergone a cycle completed to a transfer. The results of this study showed that BMI was not statistically or clinically predictive of ART outcomes or of pregnancy outcomes, among gestational carriers. It is possible that BMI alone may not be a major factor in determining the outcome of infertility treatment; other metabolic and endocrine factors may be at play [58].

The studies of the Forkhead transcription factors family (FOX) conducted in recent years are also interesting. They play an important role in regulating the expression of genes involved in cell growth, proliferation and differentiation. Studies of human endothelial cells and gene knockout mouse models have revealed the role of FOXO proteins in regulating endothelial cell angiogenic activity and blood vessel formation [59,60]. Study in loss-of-function mouse models revealed that FOXO1 significantly downregulated arterial gene expression in the mouse yolk sac prior to the onset of blood flow in early embryonic development and downregulated *Kdr* transcripts without affecting the overall identity, survival, or proliferation of endothelial cells [61]. Another member of the FOX family, FOXP3, has been reported to inhibit breast cancer angiogenesis by downregulating VEGF expression [62]. *FOXP3* gene variants and haplotypes are associated with altered incidence of RPL [5,6].

Normal angiogenesis enables the development of the placenta and a successful pregnancy. It is tightly regulated by a balance of pro- and anti-angiogenic factors that are the subject of much research. There are suggestion that infertile women with RIF could benefit from the use of platelet-rich plasma (PRP) containing growth factors (PDGF, EGF, TGFβ, VEGF, HGF, FGF2) [63].

Moreover, miRNAs are abundantly expressed in the human placenta, and miRNA dysregulation is associated with recurrent pregnancy loss and the pathogenesis of repeated implantation failures. Recently published studies indicate that miR-16 regulates angiogenesis and placental development by targeting VEGF expression and is involved in the pathogenesis of RSA [64]. In a study, Wang et al. [65], differentially analyzed the raw data deposited in microarray datasets, to screen DE-mRNAs, DE-miRNAs, and DE-circRNAs, respectively. The kinase insertion domain receptor (KDR) gene was identified by the protein–protein interaction network as one of six hub genes and was downregulated in RIF endometrial tissue samples compared to fertile control samples. In addition, three miRNAs (hsa-miR-424-5p, hsa-miR-195-5p and hsamiR-29b-3p) targeting KDR mRNA were differentially expressed in RIFs [65].

The improvement of conditions for successful implantation in patients with RIF includes the variety of strategies. It is well known that one of the important causes of RIF is the poor oocytes quality. Some interesting studies shown that the oocytes quality could be improved by myo-inositol supplementation, a compound known for its multiple role in the induction of ovulation [66]. In the case of chronic anovulation, the other form of this compound, d-chiro-inositol, was shown to modulate the activity of aromatase by reducing gene expression, inducing in this way the ovulation [67]. Some considerations focus on enhancing the implantation rate by using the embryo culture supernatant to endometrial cavity before embryo transfer [68]. Another reason of fertilization failure caused by the male factor is the cryptic sperm defects in apparently normal spermatozoa. Some studies focused on these problems indicate the necessity to conduct routine tests to detect sperm defects [69]. It is also very important to determine the role of genetic causes connected with infertility, which is suspected in at least about half of all cases. The genes involved in meiosis, DNA repair, ovarian development, steroidogenesis, folliculogenesis, and spermatogenesis could play pivotal role in fertilization failure mechanisms. On the other hand, the presence of autoimmune antibodies remains to play the role in infertile processes. Thus, cell and gene therapies could be very helpful for infertile couples to improve their autoimmune conditions and, in this way also, the oocyte maturation and embryo development [70]. Interesting also is the use of artificial intelligence algorithms for enhancing diagnosis of the RIF and ART outcome (pregnancy rate, live birth rate). The computerised analysis systems include ultrasound monitoring of folliculogenesis, endometrial receptivity, embryo selection based on quality and viability, prediction of post implantation embryo development, and oocyte and semen analysis. Through the implementation of different computer algorithms, it is possible to analyse the biological and clinical predispositions in infertile couples [71]. Relatively new are the insights of psychological variables involved in the risk condition of medically-assisted reproduction. The studies focus on depression and anxiety levels according to the number of ART attempts and, on the other hand, they assess the impact of ART on the quality of life and family interactions in couples undergoing ART procedures. These considerations could enhance mental wellbeing in infertile couples [72].

## 4. Materials and Methods

### 4.1. Patient Selection

Our study population included 247 infertile women who underwent an ART treatment cycle and were recruited into the study. All women were enrolled in the Department of Infertility and Reproductive Endocrinology of Poznan University of Medical Sciences, Poznan, Poland between January 2017 and December 2022.

Recurrent implantation failure was defined as the absence of pregnancy after three cycles of IVF using good quality embryos. All women included in the study had their own good quality embryos available for transfer. Each patient in the study group had a regular menstrual cycle and an optimal basal serum follicle stimulating hormone (FSH) level measured on the third day of the last cycle. None of the patients had been taking hormone therapy within the last three months.

The exclusion criteria for the study group were as follows: an abnormal karyotype of parents and any identified fetal genetic abnormalities, systemic connective tissue disorder, antiphospholipid antibody syndrome, hereditary thrombophilia, positive antinuclear antibodies, endocrine dysfunction (luteal insufficiency, hyperprolactinemia, thyroid diseases), and alternative reason for subfertility such as infectious and anatomical causes.

All women in the study group received luteal phase support and underwent ICSI to increase the chance of conception. Fresh or frozen embryo transfer was always performed on the fifth day, by two people (minimum 15 years of experience in the same clinic). Preimplantation Genetic Screening and Diagnosis (PGS/PGD) methods were not performed due to lack of medical indications.

One hundred and twenty age-matched, healthy women with at least two uncomplicated pregnancies ending in the live birth of a healthy full-term newborn were selected for the control group. All women from the control group without evidence of reproductive difficulty had naturally conceived pregnancies. All subjects from the control group had regular menstrual cycles, no evidence of autoimmunity and no past history of pregnancy loss or immunological and endocrinological diseases.

Patients and controls were of Polish origin, from the same geographical area. All patients were informed about the purpose of the study and gave their written consent to participation. The study was approved by the Ethics Committee of the Poznan University of Medical Sciences (no. 1159/19, date: 5 December 2019).

All procedures performed in this study were in accordance with the ethical standards of our university and with the Helsinki Declaration.

### 4.2. Sample Collection for Genetic Testing and DNA Extraction

The genomic DNA sample was stored in S-Monovette EDTA-coated tubes (Sarstedt, Nümbrecht, Germany) and extracted from peripheral blood leukocytes using the QIAamp DNA Mini Kit according to the manufacturer’s instructions (Qiagen GmbH, Hilden, Germany). DNA concentration and quality were determined spectrophotometrically using a NanoDrop 2000c spectrophotometer (Thermo Fisher Scientific, Waltham, MA, USA). Isolated DNA was stored at −80 °C until analysis. All participants signed informed consent for genetic testing, in which the study management was described.

### 4.3. DNA Amplification and Genotyping

Five SNPs, localized in the genes encoding angiogenesis-related factors, were selected according to the SNP database (dbSNP) of the National Center for Biotechnology Information (NCBI) [41] (http://www.ncbi.nlm.nih.gov/projects/SNP, accessed on 22 February 2022) and the 1000 Genomes Project data (http://www.internationalgenome.org/, accessed on 22 February 2022), based on minor allele frequency (MAF) of at least 5% in European populations. Basic information about the tested variants is presented in Table 7.

Genotyping was performed in the Molecular Biology Laboratory of Poznan University of Medical Science by polymerase chain reaction and restriction fragment length polymorphism (PCR-RFLP).

The primers and restriction enzymes used for the RFLP reactions were from previously published research and are presented in Table 2 [29,40,73,74]. Products were analyzed by electrophoresis on 2% agarose gel with Midori Green Advanced DNA Stain (Nippon Genetics, Düren, Germany). Positive and negative controls were included in each reaction and for quality control, 10% of the samples were randomly genotyped twice by different individuals, and the reproducibility was 100%. SNP characteristics, primer sequences, and details of the PCR-RFLP assays are presented in Table 8.

### 4.4. Anti-Müllerian Hormone Analyses

Blood samples were drawn from an antecubital vein between 8 a.m. and 10 a.m. after an 8 h fast into serum vacuum tubes (Becton, Dickinson and Company Franklin Lakes, NJ, USA). After the blood had clotted at room temperature for 15–30 min, the samples were centrifuged at 1000–2000× *g* for 10 min and stored at −80 °C until analyses were conducted. The serum anti-Müllerian hormone (AMH) levels for the infertile cases were measured on the cobas Modular E170 immunoanalyzer (Roche Diagnostics International Ltd., Rotkreuz, Switzerland) using the Elecsys AMH Plus (measuring range: 0.07–164 pmol/L).

### 4.5. Statistical Analysis

All statistical analyses were conducted in the R statistical software version 4.1.2 [75]. For continuous variables, normality was checked by the Shapiro–Wilk test. Normally distributed continuous variables were expressed as mean ± standard deviation (SD) and in the absence of normal distribution as median and interquartile range (IQR). Bivariate analyses were conducted with the *t*-test or the Mann–Whitney test for ordinal scales, and the chi-square test or Fisher’s exact test for nominal scales. Genotype frequency distributions and the Hardy–Weinberg equilibrium (HWE) were evaluated using the SNPassoc package [76]. Genotype distributions are shown as numbers and percentages (%).The associations between infertility and the SNP variants were evaluated by odds ratios (ORs), adjusted odds ratios (AORs), and 95% confidence intervals (95% CIs) from logistic regression. Linkage disequilibrium (LD) among the selected SNPs was calculated using Haploview v.4.2 software [77]. Interaction analyses were performed using the open source MDR software [78]. GAS (Genetic Association Study Power Calculator) was used to perform power calculations [79]. A *p* value less than 0.05 was considered significant.

## 5. Conclusions

We conducted a case–control study to investigate the relationship between genetic variation in four genes of the angiogenesis pathway with infertility and RIF in Polish females. The genetic variants selected by us have been the subject of many studies before, but not in connection with RIF. We found only one article regarding the importance of rs699947 of the *VEGFA* gene in RIF Korean women. The strength of our study is that the study population consisted of a homogenous population, which minimized other possible confounding genetic variables. Another one of the strengths of our study is the careful selection of the control group. In order to test the influence of genetic variants not only on RIF but also on infertility, we selected as controls the mothers of at least two children who became pregnant without assisted reproduction methods and did not have any miscarriages.

Since maternal age and BMI are some of the major factors contributing to implantation failure, patients in the infertile and control groups were age-matched. Unfortunately, the body mass index was significantly higher in the subjects than in the control group, but we did not observe differences in mean BMI between the RIF groups and women with less than three previous failed embryo transfers. After dividing the study group, we showed that patients with RIF were statistically significantly older than infertile NO-RIF women. Because confusion is a major issue and accounts for many discrepancies between published studies, we adjusted for maternal age and BMI in the statistical analysis of the results.

This study has several potential limitations which should be acknowledged. Embryo implantation is a very complex process dependent on many factors; therefore, it is unlikely that only single nucleotide polymorphism explains the entire susceptibility to infertility and RIF. Therefore, we performed a gene–gene interaction analysis. A combination of polymorphisms of several genes is more effective in predicting disease susceptibility. For complex analysis, it could also consider the environmental data influenced to infertility and RIF. Our study focused only on maternal genetic variants, although angiogenesis occurs in both maternal and fetal placental tissues and its genetic polymorphisms may have influenced RIF. Furthermore, the sample size of the current study was relatively small, thus, the present findings need to be confirmed in future studies with a large sample size.

Due to the biological complexity and multifactorial nature of many common diseases, single genetic variants still show poor discriminatory power for diagnosis. However, understanding the molecular mechanisms of infertility and RIF by identifying new genetic variants may be the key to developing new therapeutic strategies in the future. Molecular pharmacology is the basis of new drug development and, currently, VEGFR inhibitors have been widely used in the treatment of various tumors. However, current VEGFR inhibitors are limited to a certain extent due to limited clinical efficacy and potential toxicity, which hinder their clinical application [80]. Understanding the molecular mechanisms and function genes encoding angiogenesis-related factors in health and disease is fundamental to the development of new ways to target VEGFs and their receptors.

## Figures and Tables

**Table 1:** Clinical characteristics of infertile women and controls.

Characteristic	Cases *n* = 247	Controls *n* = 120	*p*
Age (years), mean ± SD	33.11 ± 3.51	32.50 ± 3.60	0.123
min-max	24–39	23–40	
BMI (kg/m^2^), mean ± SD	23.36 ± 4.17	20.71 ± 1.79	<0.001
Normal or Underweight (BMI ≤ 25), *n* (%)	183 (74.1)	120 (100.0)	<0.001 *
Overweight or Obese (BMI > 25), *n* (%)	64 (25.9)	0 (0.0)	
AMH (pmol/L), median (IQR)	21.00 (11.17–30.79)	—	—
Indications for ART, *n* (%)			
Idiopathic infertility	119 (48.2)	—	—
Male factor	89 (36.0)	—	
Female factor (Oviduct + Ovulatory)	39 (15.8)	—	
Number of failed transfers, *n* (%)			
1–2	175 (70.9)	—	—
RIF (≥3)	72 (29.1)	—	
Number of pregnancies, *n* (%)	188 (100.0)	293 (100.0)	<0.001 *
0	95 (38.5)	0 (0.0)	
1	120 (48.6)	0 (0.0)	
2	28 (11.3)	81 (67.5)	
≥3	4 (1.6)	39 (32.5)	
Embryo transfers, *n* (%)		—	—
Fresh Embryo Transfer	55 (29.3)		
Frozen Embryo Transfer	133 (70.7)		

(*—statistical significance).

**Table 2:** Distribution of SNPs alleles in study subjects.

SNP	Group	Major Allele *n* (%)	Minor Allele *n* (%)	HWE *p* Value	OR (95% CI)	Chi2 (df = 2)	Pearson’s p
*VEGFA* rs699947 C > A	Case	247 (0.50)	247 (0.50)	0.375	0.860 (0.632–1.172)	0.909	0.340
	Controls	111 (0.46)	129 (0.54)	0.856			
*FLT1* rs722503 T > C	Case	371 (0.75)	123 (0.25)	0.865	0.995 (0.696–1.420)	0.001	0.976
	Controls	180 (0.75)	60 (0.25)	0.809			
*KDR* rs2071559 A > G	Case	224 (0.45)	270 (0.55)	0.249	1.378 (1.011–1.877)	4.131	0.042
	Controls	128 (0.53)	112 (0.47)	0.278			
*KDR* rs1870377 T > A	Case	351 (0.71)	143 (0.29)	0.646	1.052 (0.747–1.482)	0.084	0.772
	Controls	173 (0.72)	67 (0.28)	1.000			
*FGF2* rs308395 C > G	Case	427 (0.86)	67 (0.14)	0.096	1.142 (0.717–1.819)	0.311	0.577
	Controls	211 (0.88)	29 (0.12)	0.375			

**Table 3:** Genotypic frequencies of selected polymorphisms of genes encoding angiogenesis related factors between infertile women and controls.

SNP (rs ID)/Model	Genotypes	Controls *n* (%)	Case *n* (%)	Crude			Adjusted		
				OR (95% CI)	*p*-Value	AIC	AOR (95% CI)	*p*-Value	AIC
*VEGFA* (rs699947)									
Codominant	CC	25 (20.8)	58 (23.5)	1.00	0.585	468.8	1.00	0.696	417.9
	CA	61 (50.8)	131 (53.0)	0.93 (0.53–1.62)			0.97 (0.53–1.77)		
	AA	34 (28.3)	58 (23.5)	0.74 (0.39–1.38)			0.78 (0.39–1.54)		
Dominant	CC vs. CA-AA	95 (79.2)	189 (76.5)	0.86 (0.50–1.46)	0.569	467.6	0.90 (0.51–1.60)	0.727	416.5
Recessive	CC-CA vs. AA	86 (71.7)	189 (76.5)	0.78 (0.47–1.27)	0.314	466.9	0.79 (0.47–1.35)	0.397	415.9
Overdominant	AA-CC vs. CA	59 (49.2)	116 (47.0)	1.09 (0.71–1.69)	0.692	467.7	1.11 (0.70–1.78)	0.651	416.4
log-Additive	0,1,2	120 (32.7)	247 (67.3)	1.17 (0.85–1.61)	0.328	466.9	1.14 (0.81–1.60)	0.454	416.1
*FLT1* (rs722503)									
Codominant	TT	68 (56.7)	140 (56.7)	1.00	0.998	469.9	1.00	0.928	418.5
	TC	44 (36.7)	91 (36.8)	1.00 (0.63–1.59)			1.10 (0.67–1.80)		
	CC	8 (6.7)	16 (6.5)	0.97 (0.40–2.38)			1.11 (0.43–2.88)		
Dominant	TT vs. TC-CC	52 (43.3)	107 (43.3)	1.00 (0.64–1.55)	0.998	467.9	1.10 (0.68–1.76)	0.700	416.5
Recessive	TT-TC vs. CC	112 (93.3)	231 (93.5)	0.97 (0.40–2.33)	0.945	467.9	1.07 (0.42–2.71)	0.886	416.6
Overdominant	TT-CC vs. TC	76 (63.3)	156 (63.2)	1.01 (0.64–1.58)	0.974	467.9	1.08 (0.67–1.76)	0.749	416.5
log-Additive	0,1,2	120 (32.7)	247 (67.3)	0.99 (0.70–1.42)	0.977	467.9	1.07 (0.73–1.57)	0.714	416.5
*KDR* (rs2071559)									
Codominant	AA	31 (25.8)	46 (18.6)	1.00	0.102	465.3	1.00	0.042	412.3
	AG	66 (55.0)	132 (53.4)	1.35 (0.78–2,32)			1.41 (0.78–2.55)		
	GG	23 (19.2)	69 (27.9)	2.02 (1.05–3.90)			2.42 (1.19–4.90)		
Dominant	AA vs. AG-GG	89 (74.2)	201 (81.4)	1.52 (0.91–2.56)	0.116	465.4	1.66 (0.95–2.93)	0.078	413.5
Recessive	AA-AG vs. GG	97 (80.8)	178 (72.1)	1.63 (0.96–2.79)	0.065	464.5	1.89 (1.07–3.34)	0.025	411.6
Overdominant	AA-GG vs. AG	54 (45.0)	115 (46.6)	0.94 (0.61–1.46)	0.779	467.8	0.89 (0.56–1.42)	0.625	416.4
log-Additive	0,1,2	120 (32.7)	247 (67.3)	0.70 (0.51–0.98)	0.034	463.4	0.64 (0.45–0.91)	0.013	410.4
*KDR* (rs1870377)									
Codominant	TT	62 (51.7)	123 (49.8)	1.00	0.945	469.8	1.00	0.724	418.0
	TA	49 (40.8)	105 (42.5)	1.08 (0.68–1.70)			1.21 (0.74–1.99)		
	AA	9 (7.5)	19 (7.7)	1.06 (0.45–2.49)			1.24 (0.50–3.06)		
Dominant	TT vs. TA-AA	58 (48.3)	124 (50.2)	1.08 (0.70–1.67)	0.737	467.8	1.21 (0.76–1.95)	0.422	416.0
Recessive	AA-AT vs.TT	111 (92.5)	228 (92.3)	1.03 (0.45–2.35)	0.948	467.9	1.13 (0.47–2.71)	0.784	416.5
Overdominant	AA-TT vs. AT	71 (59.2)	142 (57.5)	1.07 (0.69–1.67)	0.760	467.8	1.17 (0.73–1.89)	0.511	416.2
log-Additive	0,1,2	120 (32.7)	247 (67.3)	1.05 (0.74–1.49)	0.769	467.8	1.15 (0.79–1.68)	0.452	416.1
*FGF2* (rs308395)									
Codominant	CC	94 (78.3)	188 (76.1)	1.00	0.864	469.6	1.00	0.513	417.3
	CG	23 (19.2)	51 (20.6)	1.11 (0.64–1.92)			1.36 (0.75–2.46)		
	GG	3 (2.5)	8 (3.2)	1.33 (0.35–5.14)			1.54 (0.37–6.47)		
Dominant	CC vs. CG-GG	26 (21.7)	59 (23.9)	1.13 (0.67–1.92)	0.635	467.7	1.38 (0.79–2.43)	0.253	415.3
Recessive	CC-CG vs.GG	117 (97.5)	239 (96.8)	1.31 (0.34–5.01)	0.693	467.7	1.44 (0.35–6.02)	0.608	416.4
Overdominant	CC-GG vs. CG	97 (80.8)	196 (79.4)	1.10 (0.63–1.90)	0.739	467.8	1.34 (0.74–2.42)	0.325	415.7
log-Additive	0,1,2	120 (32.7)	247 (67.3)	1.13 (0.72–1.75)	0.595	467.6	1.31 (0.82–2.12)	0.256	415.3

AOR—*p* values adj. on age and BMI, AIC—Akaike information criterion.

**Table 4:** Clinical characteristics of infertile women divided into RIF and NO-RIF subgroups.

Characteristic	NO-RIF *n* = 175	RIF *n* = 72	*p*
Age (years), mean ± SD	32.7 ± 3.4	34.1 ± 3.7	0.005
min–max	24–39	25–39	
BMI (kg/m^2^), mean ± SD	23.6 ± 4.3	22.7 ± 3.8	0.130
Normal or Underweight (BMI ≤ 25), *n* (%)	127 (72.6)	56 (77.8)	0.491
Overweight or Obese (BMI > 25), *n* (%)	48 (27.4)	16 (22.2)	
AMH (pmol/L), median (IQR)	20.60 (11.4–29.8)	22.80 (10.7–30.9)	0.939
Indications for ART, *n* (%)			
Idiopathic infertility	84 (48.0)	35 (48.6%)	0.763
Male factor	65 (37.1)	24 (33.3%)	
Female factor (Oviduct + Ovulatory)	26 (14.9)	13 (18.1%)	
Number of pregnancies, *n* (%)	147 (100.0)	41 (100.0)	
0	53 (30.3)	42 (58.3)	<0.001
1	99 (56.6)	21 (29.2)	
2	21 (12.0)	7 (9.7)	
≥3	2 (1.1)	2 (2.8)	
Embryo transfers, *n* (%)			0.699
Fresh Embryo Transfer	44 (29.9)	11 (26.8)	
Frozen Embryo Transfer	103 (70.1)	30 (73.2)	

**Table 5:** Pregnancy outcomes of IVF embryo transfer cycles.

Characteristic	Fresh Embryo Transfers *n* = 55	Frozen Embryo Transfers *n* = 133	*p*
Pregnancy outcome, n (%)			0.532
Miscarriages	8 (14.5)	15 (11.3)	
Live births	47 (85.5)	118 (88.7)	
Mode of delivery, *n* (%)			0.015
Vaginal	26 (55.3)	41 (34.7)	
C-section	21 (44.7)	77 (65.3)	
Newborn birthweight (g), mean ± SD	3315.1 ± 512.6	3458.1 ± 412.8	0.094
Low (<2500 g), n (%)	2 (4.3)	1 (0.8)	0.435
Normal (2500–4000 g), n (%)	41 (87.2)	110 (93.2)	
Macrosomic (>4000 g), n (%)	4 (8.5)	7 (6.0)	
Placenta weight (g), mean ± SD	605.5 ± 97.5	653.5 ± 355.0	0.257
Apgar score at 1 min, median (IQR)	10.0 (10.0–10.0)	10.0 (10.0–10.0)	0.269
Apgar score at 5 min, median (IQR)	10.0 (10.0–10.0)	10.0 (10.0–10.0)	0.722
Gestational age (weeks), mean ± SD	38.2 ± 1.2	37.3 ± 5.8	0.117

**Table 6:** Genotypic frequencies of selected polymorphisms between RIF and NO-RIF infertile women.

Gene (rs ID)	Genotypes	NO-RIF *n* = 175	RIF *n* = 72	*p* Crude	AOR
*VEGFA* (rs699947)	CC	48 (27.4%)	10 (13.9%)	0.070	0.052
	CA	89 (50.9%)	42 (58.3%)		
	AA	38 (21.7%)	20 (27.8%)		
*FLT1* (rs722503)	TT	101 (57.7%)	39 (54.2%)	0.751	0.722
	TC	62 (35.4%)	29 (40.3%)		
	CC	12 (6.9%)	4 (5.6%)		
*KDR* (rs2071559)	AA	30 (17.1%)	16 (22.2%)	0.431	0.609
	AG	98 (56.0%)	34 (47.2%)		
	GG	47 (26.9%)	22 (30.6%)		
*KDR* (rs1870377)	TT	87 (49.7%)	36 (50.0%)	0.415	0.451
	TA	77 (44.0%)	28 (38.9%)		
	AA	11 (6.3%)	8 (11.1%)		
*FGF2* (rs308395)	CC	131 (74.9%)	57 (79.2%)	0.770	0.790
	CG	38 (21.7%)	13 (18.1%)		
	GG	6 (3.4%)	2 (2.8%)		

AOR—*p* values adj. on age and BMI.

**Table 7:** Primary information of genotyped SNPs.

Gene	rs No.	Location *	Base Change	MAF **
*VEGFA*	rs699947	chr6:43768652	C > A	A = 0.4950
*FLT1*	rs722503	chr13:28422915	T > C	C = 0.2435
*KDR*	rs2071559	chr4:55126199	A > G	G = 0.4851
*KDR*	rs1870377	chr4:55106807	T > A	A = 0.2346
*FGF2*	rs308395	chr4:122825787	C > G	G = 0.1630

* Location on chromosome based on human reference sequence (GRCh38.p13); ** MAF, minor allele frequency (1000 Genomes project, EUR samples).

**Table 8:** PCR-RFLP assays used in the genotyping of study SNPs.

Gene	rs No	Primer Sequences	Restriction Enzyme	Fragment Lenght (bp)
*VEGFA*	rs699947	5′- GGATGGGGCTGACTAGGTAAGC-3′ 5′- AGCCCCCTTTTCCTCCAAC-3′	BglII	C 324 A 202, 122
*FLT1*	rs722503	5′- TCCGCCTGCATTTTGAACAACTAAGTAG-3′ 5′- GGTCTCCTTGGTATTCAAGCACACGTAA-3′	AvaII	T 368 C 199, 169
*KDR*	rs2071559	5′- CAAACTTTCACTAGGGCTCTTCGT-3′ 5′- AGCCACAAGGGAGAAGCGGATA-3′	BsmI	A 290 G 174, 116
	rs1870377	5′- GCCTCACATATTATTGTACCATCC-3′ 5′- CCTCCTGTATCCTGAATGAATCT-3′	AluI	T 213, 191 A 404
*FGF2*	rs308395	5′- TGAGTTATCCGATGTCTGAAATG-3′ 5′- TAACTTGAATTAGACGACGCAGA-3′	BseNI (BsrI)	C 437 G 369, 69

bp—base pair.

## Data Availability

Not applicable.
